# Recent developments in eosinophilic renal neoplasms: what's new, true and important?

**DOI:** 10.1111/his.70001

**Published:** 2025-12-12

**Authors:** Kiril Trpkov, Farshid Siadat, Rola Saleeb

**Affiliations:** ^1^ Diagnostic and Molecular Pathology, Cumming School of Medicine University of Calgary Calgary AB Canada; ^2^ Department of Laboratory Medicine and Pathobiology University of Toronto Toronto ON Canada

**Keywords:** emerging renal entity, eosinophilic renal tumour, kidney, new renal entity, RCC, renal cell carcinoma

## Abstract

We focus in this review on the latest developments on several eosinophilic renal entities, aiming to provide an update on this topic that was previously addressed by the Genitourinary Pathology Society in their consensus papers on existing renal entities, and on novel, emerging, and provisional renal entities, and in the World Health Organization 2022 Classification of Renal Cell Tumours (5th Edition). The scope of this review includes an update on more recently described eosinophilic renal entities, including low‐grade oncocytic renal tumour (LOT), eosinophilic vacuolated tumour (EVT), folliculin (*FLCN*) mutated tumour, succinate dehydrogenase (SDH)‐deficient renal cell carcinoma, epithelioid angiomyolipoma/epithelioid PEComa (eAML/ePEComa), eosinophilic solid and cystic renal cell carcinoma (ESC RCC), anaplastic lymphoma kinase (ALK)‐rearranged RCC, fumarate hydratase (FH)‐deficient RCC, papillary renal neoplasm of reversed polarity (PRNRP), tubulocystic RCC (TC‐RCC), and thyroid‐like follicular carcinoma of kidney (TLFCK). These renal entities fall within the spectrum of eosinophilic renal tumours, in addition to the more common ones with eosinophilic features that will not be covered in this review, such as clear cell renal RCC, papillary RCC, chromophobe RCC, TFE3 rearranged RCC, and TFEB‐altered RCC. Pathologists need to consider these less common renal entities in the differential of any eosinophilic renal tumour to be able to diagnose them for the benefit of their patients. The recent developments and acquired knowledge on newer renal entities with eosinophilic cytoplasm opened insights into the clinical, pathological, immunohistochemical, molecular, epidemiological aspects, and the prognosis of these entities. We emphasize the role of routine morphology, aided by appropriate and select immunohistochemistry, as essential keys for diagnosing eosinophilic renal tumours.

## Graphical abstract

Recent developments and acquired knowledge on newer renal entities with eosinophilic cytoplasm opened insights into the clinical, pathological, immunohistochemical, molecular, epidemiological aspects, and the prognosis of these entities. We emphasize the role of routine morphology, aided by appropriate and select immunohistochemistry, as essential keys for diagnosing such eosinophilic renal tumours.

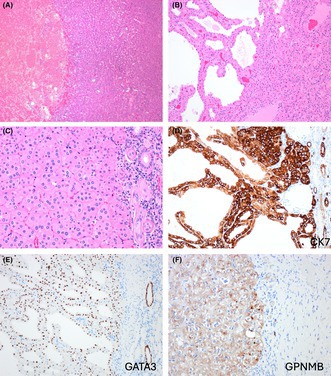

**Abbreviations**
aCGH: array‐based comparative genomic hybridization
ALK: anaplastic lymphoma kinase
AMACR: α‐methylacyl‐CoA racemase
AML: angiomyolipoma
BHD: Birt–Hogg–Dubé
ESC: eosinophilic solid and cystic
EVT: eosinophilic vacuolated tumour
FH: fumarate hydratase
*FLCN*
: folliculin
GUPS: Genitourinary Pathology Society
HLRCC: hereditary leiomyomatosis and renal cell carcinoma
IHC: immunohistochemical
LOT: low‐grade oncocytic tumour
NGS: next‐generation sequencing
PRNRP: papillary renal neoplasm of reverse polarity
RCC: renal cell carcinoma
RO: renal oncocytoma
SDH: succinate dehydrogenase
SNP: single‐nucleotide polymorphism
TLFCK: thyroid‐like follicular carcinoma of kidney
TMB: tumour mutational burden
TSC: tuberous sclerosis complex
WHO: World Health Organization

“When a thing is new, people say: ‘It is not true.' Later, when its truth becomes obvious, they say: ‘It is not important.' Finally, when its importance cannot be denied, they say: ‘Anyway, it is not new.'” William James (1842–1910), https://www.azquotes.com/quote/460303

## Introduction

Renal tumours in general, and particularly eosinophilic (pink) renal tumours, exhibit broad biological complexity and remain a fascinating and challenging topic in pathology that has undergone rapid changes in the last 10–15 years. In this review, we focus on the latest developments on several eosinophilic renal entities, particularly the most recent evidence since the consensus updates by the Genitourinary Pathology Society (GUPS) on existing renal entities^1^ on novel, emerging, and provisional renal entities,^2^ and the publication of the World Health Organization (WHO) 2022 Classification of Renal Cell Tumours (5th Edition).^3^ The scope of this review will focus only on the more recently described entities and not the common ones, such as clear cell renal cell carcinoma (RCC), papillary RCC, chromophobe RCC (classic), as well as TFE3‐rearranged RCC and TFEB‐altered RCC, all of which commonly exhibit eosinophilic features. These recent advances opened new insights into clinical, pathological, immunohistochemical (IHC), molecular, epidemiological aspects, and the prognosis of renal entities with eosinophilic cytoplasm.

From a morphological standpoint, we have grouped the entities included in this review based on their dominant morphology, as those with predominantly^1^ solid growth, and those with^2^ papillary and/or tubulocystic growth, as given in Table 1. Such morphological groups can also be used when considering the differential, during the work‐up of eosinophilic renal tumours in practice. Regarding their clinical behaviour, the eosinophilic renal entities can be stratified based on their risk for clinical progression, into those with^1^ low risk,^2^ intermediate risk, and^3^ high risk for progression, as presented in Table 2.

**Table 1:** Eosinophilic renal tumours grouped based on their dominant morphology

Eosinophilic renal tumours—Dominant morphology	
Solid	Papillary and/or tubulocystic
Low‐grade oncocytic tumour (LOT)	Eosinophilic solid and cystic (ESC) RCC
Eosinophilic vacuolated tumour (EVT)	ALK‐rearranged RCC
Folliculin (*FLCN*) mutated tumour	Fumarate hydratase (FH)‐deficient RCC
Succinate dehydrogenase (SDH)‐deficient RCC Epithelioid angiomyolipoma (E‐AML)	Papillary renal neoplasm of reversed polarity (PRNRP)
	Tubulocystic RCC
	Thyroid‐like follicular carcinoma of kidney

**Table 2:** Eosinophilic renal tumours stratified based on their risk for clinical progression

Eosinophilic renal tumours—Risk stratification		
Low‐risk	Intermediate risk	High‐risk
Low‐grade oncocytic tumour (LOT) Eosinophilic vacuolated tumour (EVT) Folliculin (*FLCN*) mutated tumour SDH‐deficient RCC (usual type)	Eosinophilic solid and cystic (ESC) RCC *ALK*‐rearranged RCC Epithelioid angiomyolipoma (E‐AML) Thyroid‐like follicular carcinoma of kidney	FH‐deficient RCC SDH‐deficient RCC (high‐grade/dedifferentiated)
Papillary renal neoplasm of reversed polarity (PRNRP)		
Tubulocystic RCC		

Traditional approach in renal tumour classification includes four major criteria: (1) morphology (or a set/constellation of morphologies), (2) reproducible (or specific) IHC profile, (3) consistent molecular/genetic profile, and (4) expected biological behaviour. Prior classifications, including the recent 2022 WHO classification, have consistently used such criteria in classifying renal tumours, resulting in a meaningful and more accurate renal tumour diagnosis that reflects current evidence and knowledge, thus providing better patient treatment and prognosis.^3^, ^4^ More recently, a broader application of novel molecular techniques, including next‐generation sequencing (NGS), such as DNA sequencing and RNA fusion techniques, allowed for an in‐depth study and better understanding of the underlying molecular pathogenesis and pathways involved in renal tumour oncogenesis. However, routine morphological assessment on haematoxylin–eosin, aided by appropriate and select IHC evaluation, remains key for diagnosing renal tumours, including emerging and novel tumours with eosinophilic morphology. Such approach allows routine morphology‐based evaluation to play a pivotal role for the diagnosis of a great majority of eosinophilic renal tumours in practice, including in more resource limited environments. In fact, pathologists' work was crucial in characterizing and introducing these novel renal entities. The key features of the entities covered in this review are summarized in Table 3.

**Table 3:** Summary of key features of more recently described eosinophilic renal entities

Type	Clinical features	Morphology	IHC	Molecular features	Prognosis
Low‐grade oncocytic tumour (LOT)	Older patients (60–70 year), solitary, may be multiple in TSC	Solid, oncocytic look, paucicellular loose stromal areas, low‐grade nuclei w/ focal perinuclear halos	CD117−, CK7+, GATA3+, L1CAM+, GPNMB+	Mutations in *MTOR* (50%), *TSC1* (20%) *TSC2* (9%), *RHEB* (8%)	Benign
Eosinophilic vacuolated tumour (EVT)	Patients a decade younger than LOT (45–55 years), solitary, may be multiple in TSC	Solid, eosinophilic cells w/ large cytoplasmic vacuoles, high‐grade nuclei w/prominent nucleoli	CD117+, CK7+/CK20+ (rare cells), cathepsin K+ (often focal), GPNMB+	Mutations in *MTOR* (43%), *TSC2* (43%), *TSC1* (14%)	Benign (only one case reported w/ MS disease)
*FLCN*‐mutated tumour	In BHD patients, rarely sporadic	Solid, mosaic pattern of eosinophilic and clear cells (‘hybrid’ features), low‐grade nuclei; rare atypical cases	CD117+ (focal), CK7+ (focal), cathepsin K+, SOX9+, GPNMB+	*FLCN* mutations (rare atypical cases w/additional mutations)	Benign, rare aggressive cases (e.g. w/ TFEB amplification)
SDH‐deficient RCC	≤0.2% of all RCCs, young adults; w/ GIST or paraganglioma in the family (rarely)	Solid, low‐grade eosinophilic, rare cytoplasmic vacuoles; rare cases w/ variant and high‐grade morphology	SDHB−, CD117−, CK7−, GATA3+, L1CAM+	*SDHB* mutations (uncommon *SDHA, SDHC* or *SDHD* mutations)	Benign if low‐grade, adverse if high‐grade (70% w/ MS disease)
Epithelioid angiomyolipoma /epithelioid PEComa (eAML/ePEComa)	Sporadic or in TSC (multiple)	≥80% epithelioid cells, eosinophilic or clear cytoplasm, may show atypia	Cathepsin K+, GPNMB+, pan‐keratin−, PAX8−	*TSC/MTOR* mutations, rare PEComas w/ Xp11.2 translocations (TFE3+)	Great majority benign, 5% w/ MS–enriched for *TP53*, *RB1*, *ATRX*
Eosinophilic solid and cystic RCC	Mostly females, sporadic, 5% in TSC patients	Solid and cystic, variable patterns, voluminous eosinophilic cells, cytoplasmic stippling	CK20+ (85%), CK7−, CD117−, cathepsin K+ (focal), GPNMB+, vimentin+	Somatic bi‐allelic loss or mutation in *TSC2* and less often in *TSC1*	Majority benign, 5–10% w/ MS (may respond to MTOR‐inhibitors)
Anaplastic lymphoma kinase‐rearranged RCC	Broad age range, sickle cell trait in children	Variable admixed patterns, often w/background and/or intracellular mucin	ALK1+, PAX8+	*ALK* rearrangement w/various fusion partners	Majority benign, ≤30% w/ MS (may respond to ALK inhibitors)
FH‐deficient RCC	Some w/HLRCC syndrome	Diverse patterns, often papillary and tubulocystic, macronucleoli, rare cases w/ low‐grade oncocytic morphology	FH−, 2SC+, AKR1B10+	Germline or rarely somatic *FH* mutations	Aggressive tumours
Papillary renal neoplasm of reverse polarity (PRNRP)	Small, sporadic tumours (often ≤15 mm), can be multiple	Delicate papillae, w/single layer of oncocytic cells, luminal nuclear orientation (‘reverse polarity’)	GATA3+, L1CAM+, AMACR−	*KRAS* mutations	Benign
Tubulocystic RCC	Sporadic, solitary, <1% of all RCCs	Bubble wrap gross appearance, pure tubulocystic growth, eosinophilic cells w/prominent nucleoli	FH+, AMACR−, CK7−, CD10−	Loss of chr. 9, Y and gain of chr. 17, but specific mutations not found	Benign (great majority)
Thyroid‐like follicular carcinoma of kidney	Broad age range, no specific clinical features	Follicular pattern, follicles w/ dense eosinophilic colloid, resembles follicular thyroid carcinoma	TTF1−, thyroglobulin−, PAX8+	*EWSR1*::*PATZ1* fusion found in three cases	Usually benign, rare cases w/ MS (some w/ sarcomatoid change)

ALK, Anaplastic lymphoma kinase; FH, Fumarate hydratase; GIST, Gastrointestinal stroma tumour; HLRCC, Hereditary leiomyoma renal cell carcinoma; MS, Metastasis; RCC, Renal cell carcinoma; SDH, Succinate dehydrogenase; TSC, Tuberous sclerosis complex; w/, With.

Finally, the process of recognition of any novel renal entity, including the proposed nomenclature, will be established (or not) through independent external validations and by the acceptance of new diagnostic categories in routine practice. This process is however not straightforward, and it is to be expected that it will generate a healthy and hopefully productive debate whether any entity is ‘new, true, and important’.^4^, ^5^, ^6^, ^7^

## Low‐Grade Oncocytic Tumour (LOT)

WHO 2022 classification created a subcategory of ‘other oncocytic tumours of the kidney’,^8^ to account for a heterogeneous group of oncocytic tumours not classifiable either as renal oncocytoma (RO)^9^ or chromophobe RCC (ChRCC),^10^, ^11^, ^12^ the main entities in the category ‘oncocytic and chromophobe renal tumours’. This group has been a subject of extensive investigations in the past, and more recently its scope has been clarified further, primarily because of the recognition of two distinct eosinophilic renal entities, low‐grade oncocytic tumour (LOT)^8^, ^13^, ^14^, ^15^ and eosinophilic vacuolated tumour (EVT).^8^, ^15^, ^16^, ^17^

Since the initial descriptions of LOT in 2019,^13^, ^14^ multiple retrospective and prospective, single institution and multi‐institutional studies have fully validated the seminal findings, confirming that LOT is a unique and distinct novel renal entity.^18^, ^19^, ^20^, ^21^, ^22^, ^23^, ^24^, ^25^, ^26^, ^27^, ^28^, ^29^, ^30^, ^31^, ^32^ LOT shows overlapping morphological features between RO and eosinophilic variant of ChRCC (e‐ChRCC), with IHC profile that includes diffuse‐positive CK7 and typically negative or rarely weakly positive CD117 (KIT) (CK7+/CD117‐ profile). LOT has likely been previously misdiagnosed, for example, as RO,^22^ e‐ChRCC,^11^, ^12^ or it has been labelled descriptively as ‘oncocytic/eosinophilic renal neoplasm/tumor, not further classified/or unclassified’.^1^ LOT is typically a single, sporadic tumour,^13^, ^14^ but has been rarely found as multiple LOTs in patients with end‐stage kidney disease^22^, ^33^ and tuberous sclerosis complex (TSC).^19^, ^21^, ^34^, ^35^, ^36^ LOT was found in patients of broad age range, but usually older, around the age of 60, and more frequently in females (M:F = 1:2). Importantly, all reported LOT cases to date (>300) have demonstrated an indolent behaviour, supporting the conclusion that this novel entity is essentially a benign tumour.^14^, ^19^, ^20^, ^21^, ^22^, ^23^, ^24^, ^25^, ^26^, ^27^, ^28^, ^29^, ^30^, ^31^, ^32^ Although LOT represents less than 0.5% of all renal tumours, its reported frequency within the group of ‘eosinophilic/oncocytic renal tumors’ has been 3.7%–5.7%.^26^, ^28^, ^31^

**Figure 1:**
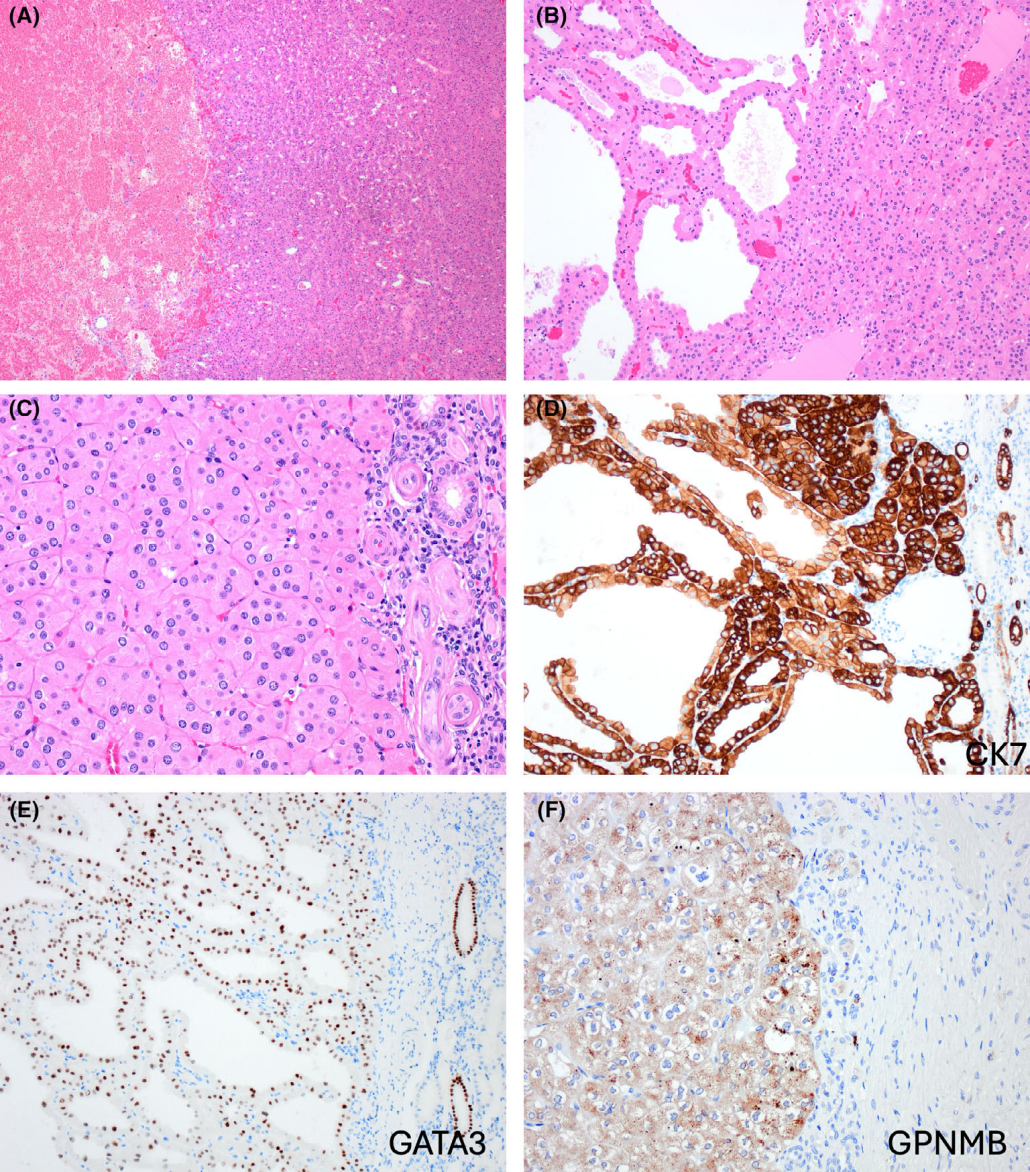
Low‐grade oncocytic tumour (LOT). (**A**) LOT shows solid growth (right) with interspersed loose stromal areas (left), often with haemorrhage and containing cells arranged in irregular cords and as individual cells (‘boats in a bay’). (**B**) In addition to solid growth (right), tubular, trabecular and tubulo‐papillary morphology (left) can be focally seen. (**C**) LOT lacks a peripheral capsule, and the cells are eosinophilic (‘oncocytic’) with round to oval ‘low‐grade’ nuclei, lacking notable irregularities, with focal or more extensive perinuclear halos. (**D–F**) On IHC, CK7 is diffusely positive (**D**), as well as GATA3 (**E**) and GPNMB (**F**), while CD117 is negative (not shown).

LOT is typically a small tumour with mahogany‐brown to tan cut surface, with solid growth, as well as focal tubular, tubuloreticular, trabecular, and rarely papillary architecture (Figure 1A,B).^4^, ^14^, ^22^, ^32^ LOT shows interspersed edematous stromal areas, often with haemorrhage, containing scattered or irregularly arranged cells and cell cords (‘boats in a bay’ or ‘tissue culture’ arrangement).^2^, ^14^ The cells have homogeneous eosinophilic (‘oncocytic’) cytoplasm with round to oval ‘low‐grade’ nuclei, lacking notable irregularities, and showing either focal or more extensive perinuclear clearings (halos) (Figure 1C).

In addition to the consistent CK7+/CD117− IHC profile,^14^ LOT is also positive for GATA3^27^, ^37^ and L1CAM,^23^ both markers of distal nephron differentiation, suggesting a principal cell of distal nephron derivation (Figure 1D,E). In contrast, FOXI1, typically expressed in both RO and ChRCC, and also marking the intercalated cells of the distal tubules in the normal kidney, has been found on IHC to be negative or of very low reactivity in LOT.^19^, ^38^, ^39^ Thus, it has been proposed that LOT should be renamed as ‘oncocytic principal cell adenoma of the kidney’.^23^ While such proposal may be reasonable regarding the putative cell of origin, a longer and more complex name would potentially create confusion among pathologists, who seem to have already adopted LOT as an easy name to remember that has been broadly used in validation studies. LOT has also been shown to express (at least focally) p‐S6, p‐4EBP1, both markers associated with mTOR pathway activation,^21^ and other similar markers, such as p‐mTOR, although with variable and/or focal reactivity.^24^ GPNMB is another marker associated with TSC‐mTOR pathway activation that has been found to be uniformly and consistently positive in LOT (Figure 1F).^24^, ^40^ GPNMB is however non‐specific, because it essentially reflects the underlying pathogenetic alterations in other tumours, such as TFE3 RCC and TFEB RCC, as well as in angiomyolipoma (AML), and other TSC‐mTOR pathway associated renal tumours (for example, EVT, eosinophilic solid and cystic (ESC) RCC, and RCC with fibromyomatous stroma). On electron microscopy, the cells in LOT show abundant, closely packed and normal‐appearing mitochondria, similar to oncocytoma and EVT,^41^ and also show intracytoplasmic lumina with microvilli that are different from those observed in ChRCC.^23^

Great majority of LOTs exhibit mTOR pathway gene alterations.^18^, ^19^, ^20^, ^21^, ^23^, ^24^, ^25^, ^27^, ^28^, ^37^ Analysis of the reported molecular data of 100 LOT cases from multiple studies,^18^, ^19^, ^20^, ^21^, ^23^, ^24^, ^25^, ^28^, ^37^ showed that alterations of mTOR pathway genes were directly involved in 87% (87/100) cases, with the most frequent mutations in *MTOR* (50%, 50/100), followed by *TSC1* (20%, 20/100), *TSC2* (9%, 9/100), and *RHEB* (8%, 8/100). Other reported gene alterations included *PIK3CA, NF1, NF2, PTEN, NOTCH1, NOTCH4, CDK2A, EZH2, SETD2*, and *ATM*, which may potentially act as upstream or downstream effectors of the mTOR pathway.^23^, ^24^, ^25^, ^28^, ^37^ It has been postulated that some of these alterations, such as *PIK3CA*, may be sufficient on their own to activate the mTOR pathway.^24^, ^27^, ^28^ Of note, LOT typically demonstrates a low tumour mutational burden (TMB)^24^, ^27^ and lacks complete chromosomal gains or losses and *CCND1* rearrangements.^22^ LOT has also been reported with deletions at 19p13, 1p36, and 19q13.^14^

## Eosinophilic Vacuolated Tumour (EVT)

The first descriptions of EVT were by He *et al*. in 2018, who initially used the term ‘high‐grade oncocytic tumor (HOT)’,^16^ followed by Chen *et al*. in 2019,^17^ who used the term ‘sporadic RCC with eosinophilic and vacuolated cytoplasm’. Subsequently, the name ‘eosinophilic vacuolated tumor’ (EVT) was proposed by the GUPS consensus in 2021^2^ that was adopted in the 2022 WHO classification, where EVT was included in the section ‘other oncocytic tumours of the kidney’.^8^ Like LOT, EVT has also emerged from the group of tumours that share ‘hybrid’ morphological and IHC features of RO and ChRCC.^1^, ^42^, ^43^

To date, about 60 EVT cases have been reported in the English literature in several institutional or multi‐institutional series and as case reports, and all cases have behaved indolently.^2^, ^16^, ^17^, ^20^, ^31^, ^41^, ^44^ However, a 14‐year‐old male has been reported recently with an 11.2 cm EVT that progressed with mediastinal lymph node metastasis after 54 months, but no further progression was reported.^45^ The reported frequency among ‘eosinophilic/oncocytic renal tumors’ was 0.8%.^31^ The lower number of reported EVT cases and the lower frequency of EVT among ‘eosinophilic/oncocytic renal tumours’ indicate that EVT is less common than LOT. Although EVT was found in patients of broad age range, the reported mean age was between 44 and 55 years, about a decade younger than in patients with LOT.^16^, ^17^, ^20^, ^31^, ^44^ EVT is also slightly more frequent in women (M:F = 1:2.5).^16^, ^17^, ^20^, ^31^, ^44^ EVT has also been found in patients with TSC,^34^, ^36^, ^46^ although in some series predating the contemporary nomenclature, EVT has been likely reported with descriptive names (for example, ‘chromophobe‐like’).^47^

**Figure 2:**
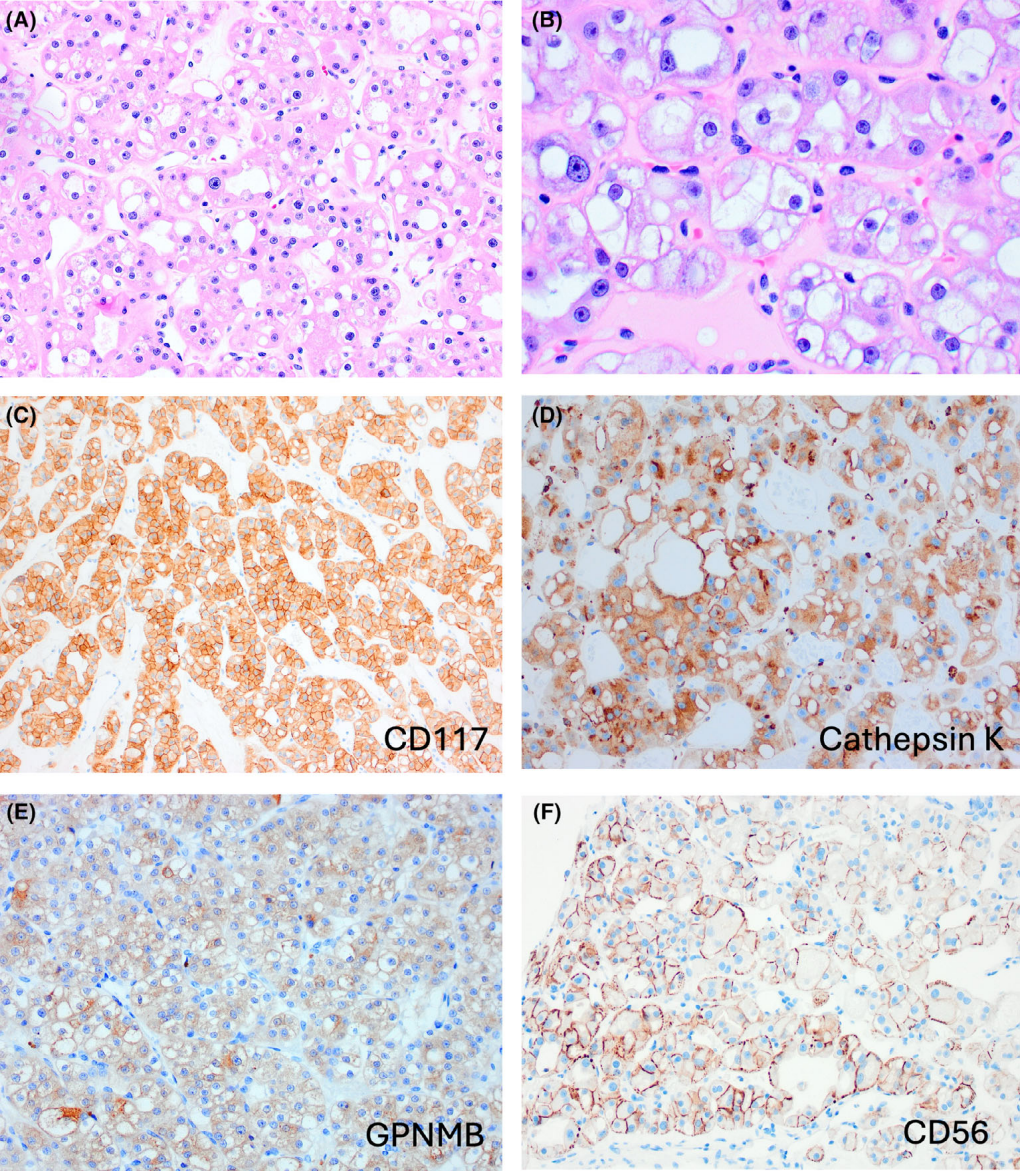
Eosinophilic vacuolated tumour (EVT). (**A**, **B**) EVT is composed of eosinophilic cells with large intracytoplasmic vacuoles and round to oval nuclei with prominent (“high‐grade”). (**C–F**) On IHC, the cells are positive for CD117 (KIT) (**C**), at least focally for cathepsin K (**D**), and GPNMB (**E**); CD56 also seems to be a sensitive marker for EVT, but not a specific one (**F**).

EVT is typically a solitary, sporadic, tan to brown tumour of smaller size, with the great majority measuring <5 cm in size.^2^, ^16^, ^17^, ^20^, ^34^, ^36^, ^44^, ^46^, ^48^ EVT is typically a solid tumour that lacks a cystic component and a well‐formed capsule, but thick‐walled vessels are commonly found at the periphery. The cells have eosinophilic cytoplasm and large intracytoplasmic vacuoles that produce cytoplasmic clearing, with round to oval nuclei with prominent (‘high‐grade’) nucleoli that may focally resemble viral inclusions (Figure 2A,B).^16^, ^17^, ^44^

EVT is consistently reactive for CD117 (KIT), CD10, and at least focally for cathepsin K in the great majority of cases, while CK7 is positive only in scattered cells (Figure 2C,D).^16^, ^44^ The IHC profile ‘CD117+/CK7+ in rare cells’ resembles the IHC profile of RO. Rare positive cells for CK20 may also be found.^16^, ^44^ GPNMB has also been found positive in EVT (Figure 2E).^40^ We also found that CD56 is consistently positive in EVT, but it is likely not specific (manuscript in preparation) (Figure 2F). On electron microscopy, EVT shows abundant intracytoplasmic mitochondria and dilated cisterns of rough endoplasmic reticulum.^41^, ^48^

*TSC/MTOR* mutations that lead to mTORC1 activation have been consistently found in EVT,^17^, ^20^, ^44^, ^48^ typically as non‐overlapping mutations associated with low TMB rates.^44^ In 35 EVT cases with reported molecular data, the frequency of *TSC/MTOR* mutations was as follows: *MTOR* (43%, 15/35), *TSC2* (43%, 15/35), and *TSC1* (14%, 5/35).^17^, ^20^, ^44^ Despite the commonality of TSC/MTOR mutations, the principal component analysis based on differentially expressed genes showed that EVT, LOT, and ESC RCC were segregated from each other and were distinct from other renal tumour types^20^ EVT also lacked complete losses or gains of chromosomes, but a loss of the wild‐type allele on chromosome 1 (where MTOR resides) has been found^17^, ^44^, ^48^ as well as isolated losses of chromosomes 19p, 16p11, and 7q31^16^, ^44^

## FLCN‐Mutated Tumours

Remaining ‘oncocytic’ renal tumours that do not fit into RO, e‐ChRCC, LOT and EVT, in the current practice are a significantly reduced group. In a sporadic setting, such tumours may be labelled descriptively ‘oncocytic renal neoplasms of low malignant potential, not further classified’.^1^, ^42^, ^49^ However, if associated with a recognized hereditary syndrome, for example, Birt–Hogg–Dubé (BHD) syndrome,^50^ renal oncocytosis,^4^, ^51^, ^52^, ^53^ or other hereditary syndromes, typically occurring as multifocal/bilateral tumours, such tumours with mixed features can be labelled ‘hybrid oncocytic chromophobe tumours’.^1^, ^4^, ^42^, ^43^ The term ‘hybrid’ in this context refers strictly to the overlapping morphological features (‘oncocytic chromophobe’) of such low‐grade ‘oncocytic’ tumours.^1^, ^4^

**Figure 3:**
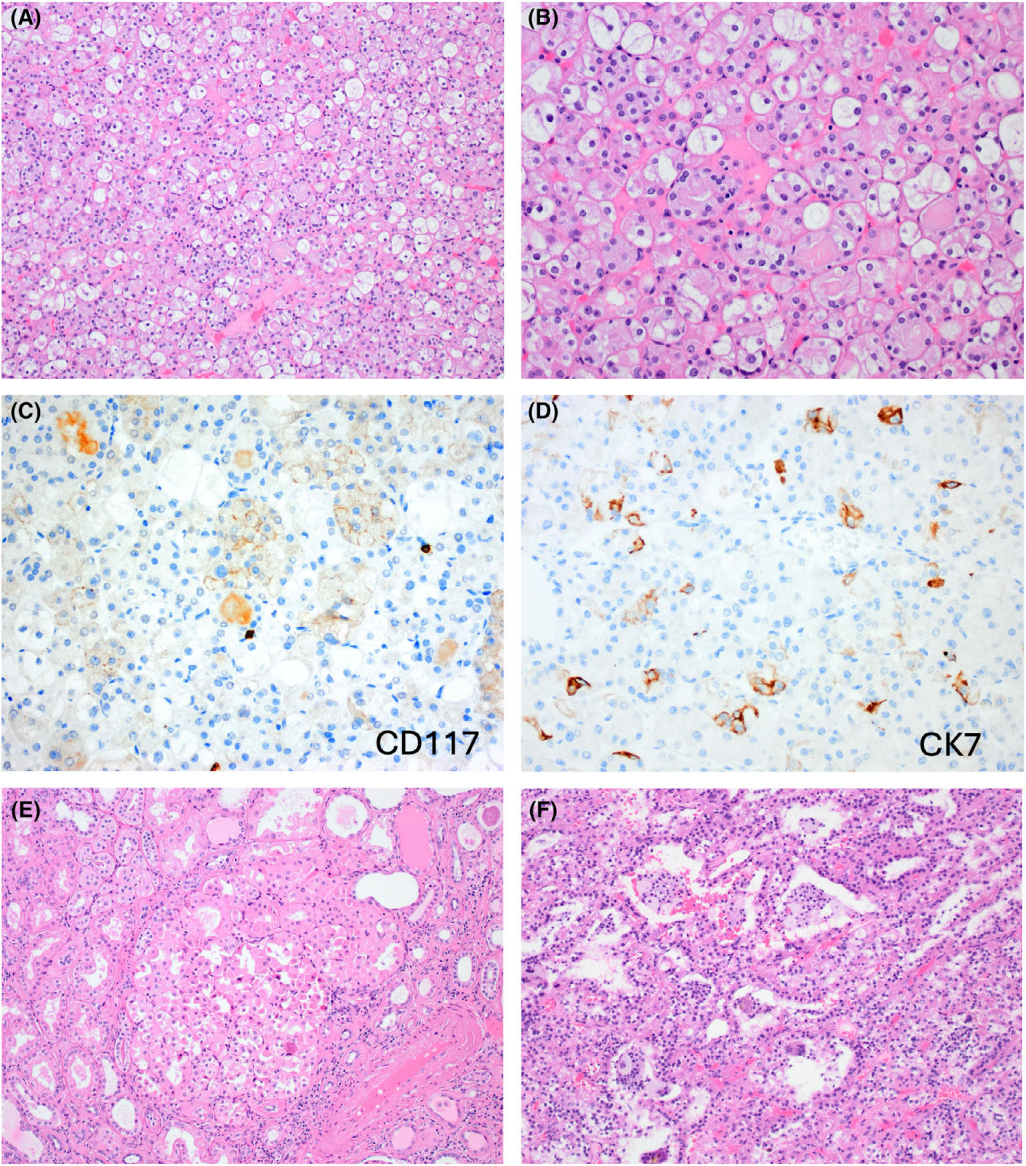
*FLCN‐*mutated tumours. (**A**, **B**) On morphology, they typically show a mosaic (“checkerboard”) pattern, with eosinophilic/oncocytic cells intermixed with variable clusters of clear cells or cytoplasmic vacuoles. (**C**, **D**) *FLCN*‐mutated tumours with such features show variable combinations of CD117 (KIT)‐reactive eosinophilic cells (**C**), and CK7‐positive clear cells (**D**). (**E**) Microscopic foci of oncocytic cells are often found in the adjacent kidney parenchyma. (**F**) A group of atypical *FLCN‐mutated* tumours has been described recently that show diverse morphologies and may exhibit additional molecular alterations. This is an example of an unclassifiable *FLCN‐*mutated tumour with eosinophilic and clear cells and dilated tubules containing histiocytes exhibiting *FLCN* p.Gln533Ter (likely oncogenic mutation) and *TSC2* p.Pro541Leu (mutation of unknown function).

Importantly, the tumours with such ‘hybrid’ features in the setting of BHD demonstrate germline mutations of the tumour suppressor gene folliculin (*FLCN*), and on morphology they typically show a mosaic (‘checkerboard’) pattern, with eosinophilic/oncocytic cells intermixed with variable clusters of cells with clear cytoplasm or cytoplasmic vacuoles (Figure 3A,B).^1^, ^42^ In addition, microscopic oncocytic cell foci are commonly present in the adjacent kidney parenchyma (Figure 3E).^50^ Such *FLCN‐*mutated tumours are invariably indolent and show benign behaviour.^53^, ^54^, ^55^, ^56^, ^57^, ^58^, ^59^, ^60^ Recent studies have shown that these tumours are indeed unique and are composed of transcriptionally distinct cell populations that are often mutually exclusive, and appear to originate from the intercalated cells and the principal cells of the distal nephron, respectively.^54^, ^61^, ^62^ The intercalated cells of the distal nephron express FOXI1 and *LINC01187*, as well as CD117 (KIT), markers uniformly expressed in RO and ChRCC, while the principal cells express L1CAM.^38^, ^54^ Thus, *FLCN*‐mutated tumours with ‘hybrids’ features also show mutually exclusive IHC features, with variable combinations of CD117 (KIT) reactive eosinophilic cells, and CK7 positive clear cells,^54^, ^59^ likely owing to their different cells of origin (Figure 3C,D). *FLCN‐*mutated tumours in BHD are also different from the sporadic ChRCC, and exhibit completely different expression profiles, as well as nuclear and mitochondrial DNA signatures.^63^ These insights question the notion of the existence of true ‘BHD‐associated ChRCC’ documented in prior studies.^59^, ^61^

On IHC, *FLCN‐*mutated BHD tumours also show frequent cathepsin K reactivity and increased expression of SOX9, in contrast to the lack of expression of SOX9 in RO, ChRCC and LOT.^53^, ^57^ Interestingly, there is a distinct group of ‘unclassified/atypical/other *FLCN* mutated tumors’,^59^, ^60^ described also as ‘non‐conventional *FLCN* mutated tumors’,^57^ that are found in BHD syndrome and also as sporadic tumours, which are potentially aggressive (Figure 3F). Such atypical *FLCN‐*mutated tumours exhibit diverse morphologies and additional molecular alterations (for example, a concomitant TFEB gain/amplification),^57^ and typically lack the mosaic (‘hybrid’) cell morphology and IHC profiles seen in the typical *FLCN‐*mutated tumours.^57^, ^59^, ^60^

All *FLCN‐*mutated tumours, including the sporadic ones and those with somatic *FLCN* mutations, appear to consistently and diffusely express GPNMB on IHC, which is reflective of the underlying mTORC1 dysregulation, leading to TFEB activation.^59^, ^60^ GPNMB reactivity is non‐specific and cannot distinguish between *FLCN‐*mutated tumours and TFE3/TFEB RCCs, as well as various other *TSC1/2/MTOR‐*mutated tumours, but it may be diagnostically useful for screening for *FLCN* mutations that would help distinguish them from their mimics.^53^, ^59^

## Succinate Dehydrogenase‐Deficient Renal Cell Carcinoma (SDH‐Deficient RCC)

After the initial descriptions, succinate dehydrogenase‐deficient (SDH‐deficient) RCC was formally recognized in the WHO 2016 classification and it was included in the latest WHO 2022 classification in the group of ‘molecularly defined renal carcinomas’.^64^, ^65^, ^66^, ^67^ SDH‐deficient RCC is a rare tumour and represents ≤0.2% of all RCCs, with over 110 cases reported to date, collected primarily through multi‐institutional collaborations.^64^, ^65^, ^66^, ^68^, ^69^ Although found in patients of broad age range, they are most common in younger adults (30–40 years), with a slight male predominance.^64^, ^65^, ^66^, ^68^, ^69^ SDH‐deficient RCC are typically solitary tumours, but bilateral and multifocal tumours have been found in 8%–30% of patients.^64^, ^69^ SDH‐deficient RCC is characterized by germline mutations of any of the four SDH genes (*SDHA*, *SDHB*, *SDHC*, and *SDHD*) and is associated with an autosomal dominant hereditary syndrome that also includes a constellation of other tumours, including phaeochromocytoma/paraganglioma, distinct gastric gastrointestinal stromal tumour, and pituitary adenoma.^70^, ^71^, ^72^, ^73^, ^74^, ^75^ A marked loss of SDH subunits results in accumulation of oncogenic succinate, which is a common, adverse, epigenetic modulating feature found, for example, in clear cell RCC associated with markedly worse overall and disease‐free survival.^76^

**Figure 4:**
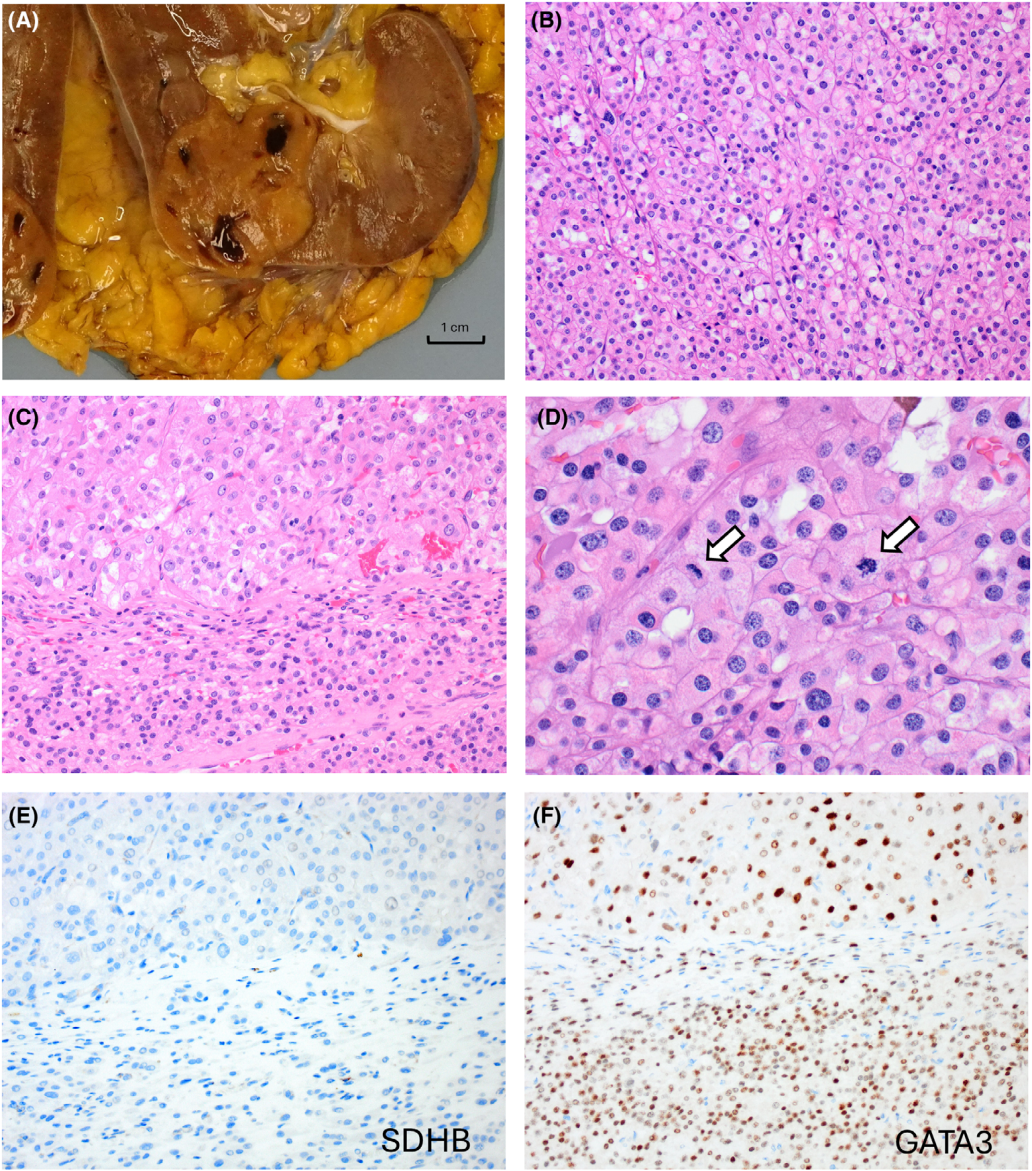
SDH‐deficient RCC. (**A**) Grossly, SDH‐deficient RCCs are solid, tan‐brown, and well circumscribed tumours. (**B**) They typically show sheets of eosinophilic cells with focal intracytoplasmic vacuoles and low‐grade nuclei. (**C**) High‐grade transformation can be found in a subset of cases that show exclusivity of only focal high‐grade areas (top), admixed with typical low‐grade areas (bottom). (**D**) Such areas may show mitotic activity (arrows), or sarcomatoid changes and necrosis (not shown). (**E**, **F**) On IHC, the tumour cells are negative for SDHB (**E**) and are positive for GATA3; the stains are illustrated in the same area, with high‐grade transformation and typical morphology shown in (**C**).

Grossly, the tumours are typically solid, with a tan or light brown to mahogany‐brown cut surface (Figure 4A). On microscopy, SDH‐deficient RCC typically shows sheet‐like or compact nested growth of bland cuboidal cells with low‐grade nuclei, eosinophilic cytoplasm and variable intracytoplasmic vacuoles (thought to represent abnormal giant mitochondria)^77^ scattered microcysts and entrapped non‐neoplastic tubules (Figure 4B,C).^64^, ^65^, ^78^ In a recent study,^69^ variant morphologies were found in 21% of cases and these included high‐grade nuclear features with prominent nucleoli and various combinations of papillary, solid, and tubular architecture, in some cases resembling fumarate hydratase (FH)‐deficient RCC and TFEB‐altered RCC.^64^, ^79^ Although most SDH‐deficient RCCs are indolent, a potential for aggressive behaviour has been found in the majority (~70%) of cases with high‐grade transformation and variant morphologies, as well as showing coagulative necrosis and sarcomatoid and rhabdoid features (Figure 3C,D).^64^, ^69^ Of note, high‐grade and sarcomatoid change in SDH‐deficient RCCs may be either focal or diffuse, resulting in ‘unclassifiable RCC’ morphology, which justifies a low threshold for IHC screening in any difficult‐to‐classify eosinophilic high‐grade renal tumour, particularly found in a younger adult.^64^, ^69^

SDH‐deficient RCC shows uniform absence of SDHB reactivity on IHC, owing to a biallelic inactivation of any of the four *SDH* genes (primarily *SDHB*, and much less frequently *SDHA*, *SHDC*, or very rarely *SDHD*), which are almost always associated with germline mutations and a somatic second hit (Figure 4E).^64^, ^70^, ^71^, ^72^, ^73^, ^74^, ^75^, ^80^ A subset of SDH‐deficient tumours can show aberrant cytoplasmic (blush to fine granular) SDHB IHC staining that can be misinterpreted as ‘retained’, which is a recognized diagnostic pitfall.^71^, ^81^, ^82^ On IHC, there is also uniform lack of reactivity for CD117 (KIT), with frequent absence or weak/focal pan‐cytokeratin and CK7 expression.^64^ Recently, it has been also shown that SDH‐deficient RCC exhibits uniform GATA3 reactivity on IHC (Figure 4F), as well as frequent L1CAM expression (72%).^83^ As both markers highlight the principal cells of the distal nephron, this may suggest that the principal cells may be the possible cell of origin, and these markers may aid the routine IHC diagnosis of SDH‐deficient RCC.^83^

Several reported SDHA‐deficient RCCs were more common in males (M:F = 4.5:1) and showed combinations of eosinophilic cells with various growth patterns, including papillary, solid, glandular, cribriform, microcystic surrounding hyaline eosinophilic globules, and desmoplastic, all of which differ from the typical morphology of SDH‐deficient RCC.^84^, ^85^, ^86^, ^87^, ^88^, ^89^ In addition to SDHB loss, IHC for SDHA is also negative (or only weakly positive) in cases with biallelic inactivation of *SDHA*.^87^, ^88^, ^89^, ^90^ Of note, some SDHA‐deficient RCCs with a concomitant *NF2* mutation and metastatic spread have responded to immunotherapy during the limited follow‐up.^88^

## Epithelioid Angiomyolipoma/Epithelioid PEComa (eAML/ePEComa)

Epithelioid angiomyolipoma/epithelioid PEComa (eAML/ePEComa) represents a variant of AML/PEComa that consists of at least 80% epithelioid cells, and it was devoted a separate section and a modified name (with addition of ‘ePEComa’) in the WHO 2022 classification.^91^ It is a notorious consideration in the differential diagnosis of many eosinophilic renal tumours, as it can mimic a RCC. In contrast to the typical triphasic AML, it is more often seen in younger patients, in TSC patients, and in patients with *TSC2/PKD1* contiguous gene syndrome, typically as a multifocal/bilateral tumour.^91^, ^92^ Although traditionally eAML/ePEComa was considered to have a higher metastatic potential, a larger multi‐institutional study on a consecutive cohort of AMLs, using a stringent 80% cutoff, has shown that only 4.6% represent true eAML, with a frequency of metastatic tumours of 5%.^93^

**Figure 5:**
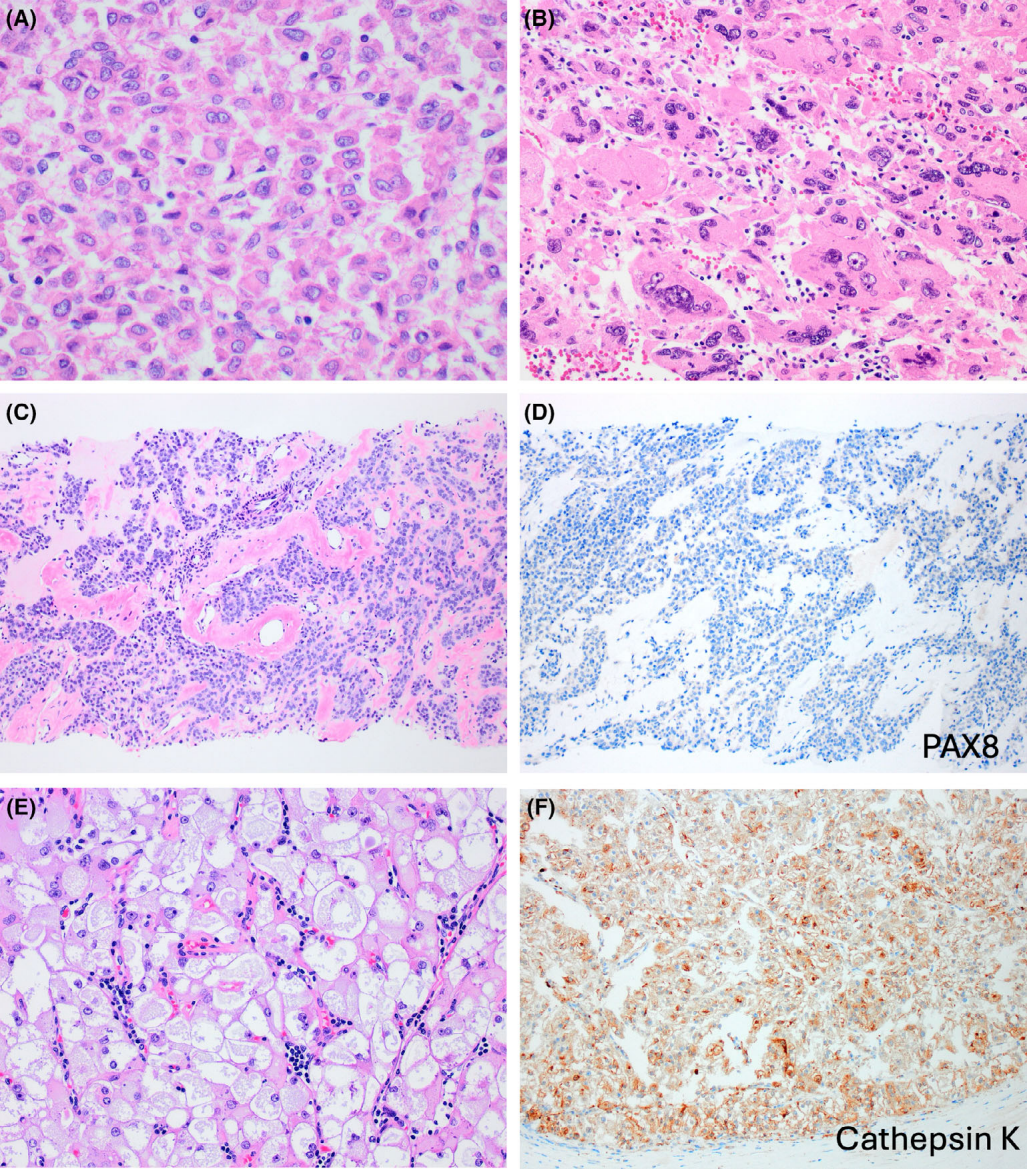
Epithelioid angiomyolipoma/epithelioid PEComa (eAML/ePEComa). (**A**) eAML/ePEComa shows carcinoma‐like morphology, with almost exclusive growth of eosinophilic epithelioid cells. (**B**) Some eAML/ePEComa can show cell atypia and pleomorphism, multinucleation, brisk mitotic activity, and necrosis that have been associated with adverse behaviour in older studies. (**C**) Cords of uniform epithelioid cells with focal perivascular arrangement embedded in a hyalinized stroma (needle core biopsy of an eAML/ePEComa in a TSC patient). (**D**) All eAML/ePEComa are negative for PAX8. (**E**, **F**) An example of TFE3‐rearranged PEComa showing voluminous clear and eosinophilic cells (**E**) and reactivity for cathepsin K, which is present in all eAML /ePEComas (**F**).

On morphology, eAML/ePEComa may show carcinoma‐like growth, with eosinophilic cells exhibiting more significant atypia and abundant cytoplasm, with prominent nucleoli and nuclear inclusions, similar to ganglion cells (Figure 5A,C).^91^ A second distinct pattern consists of more uniform epithelioid plump cells, with pale to clear, finely granular cytoplasm and monomorphic nuclei. Various morphological criteria have been proposed in the past to predict the metastatic risk in AML, including larger tumour size (more than 7 cm), necrosis, increased mitotic activity (≥2 mitoses per 10 high‐power fields) and atypical mitoses, extrarenal extension, renal vein involvement, and association with TSC (Figure 5B).^35^, ^93^, ^94^, ^95^ More recently, AMLs with metastatic behaviour were found to be enriched for alterations of *TP53*, *RB1*, *ATRX, APC*, and *NF1* genes that were found in 80% of metastatic AMLs; moreover, an IHC for *TP53*, *RB1*, *ATRX* can be used as a screening aid.^35^, ^36^, ^96^

eAML/ePEComa is negative for pankeratin and PAX8, but expresses melanocytic markers melan A and HMB45, and it is uniformly positive for cathepsin K and GPNMB (Figure 5D,F).^40^, ^91^ Smooth muscle markers (such as smooth muscle actin and desmin) are also variably expressed, but their use in the current practice is reduced. There are also exceptionally rare primary renal PEComas that harbour Xp11.2 translocations and exhibit strong and diffuse TFE3 expression by IHC, but otherwise show morphological overlap and other IHC similarities with eAML/ePEComa (Figure 5E).^97^ These findings suggest that there is likely a molecular convergence between the *TSC1/2* gene mutation‐driven mTOR activation and *TFE3* fusion‐driven MiTF pathway activation at the pathobiological level, at least in some eAML/ePEComas.^97^, ^98^

## Eosinophilic Solid and Cystic Renal Cell Carcinoma (ESC RCC)

**Figure 6:**
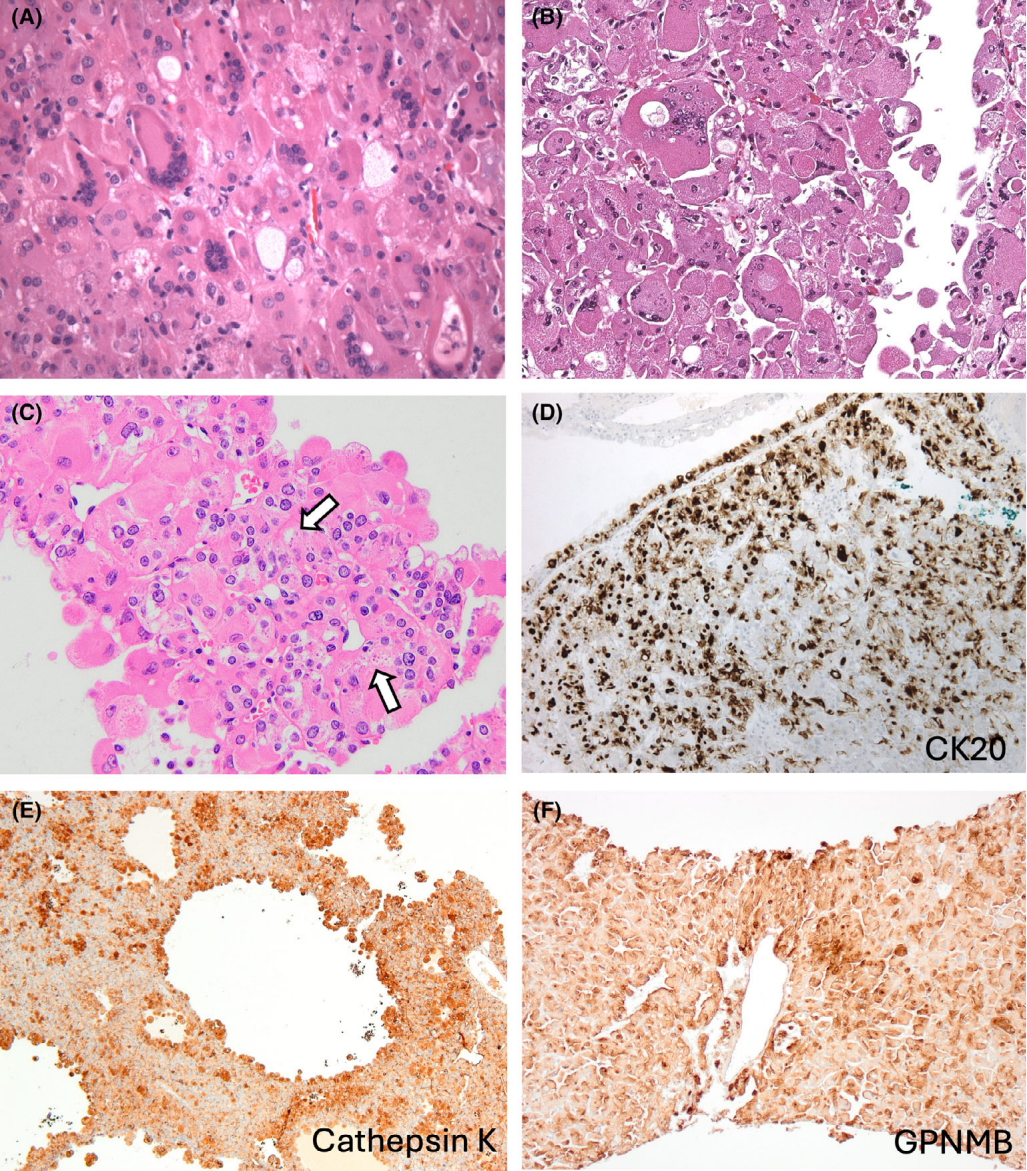
Eosinophilic solid and cystic (ESC) RCC. (**A**, **B**) ESC RCC shows cystic and solid areas composed of eosinophilic cells exhibiting diffuse and compact nested growth, but other patterns and cell morphologies may also be seen, including multinucleated and xanthomatous giant cells. (**C**) Cysts are lined by eosinophilic hobnailed cells. One of the most helpful diagnostic features is the presence of basophilic to amphiphilic coarse cytoplasmic granules (stippling) (arrows). (**D**, **F**) On IHC, ESC RCC typically shows diffuse or focal CK20 expression in the majority of cases (**D**), as well as cathepsin K (**E**) and GPNMB reactivity (**F**).

ESC RCC was recognized as a novel renal entity in the WHO 2022 classification and was included in the category ‘other renal tumours’.^15^, ^99^ ESC RCC was likely previously diagnosed as ‘unclassified RCC with oncocytic/eosinophilic features’ or it has been misdiagnosed as other entities with eosinophilic features, such as papillary RCC or ChRCC.^18^, ^20^, ^100^, ^101^ Cases historically considered ‘oncocytoid renal cell carcinomas after neuroblastoma’ or occurring after other childhood malignancies have also been recently shown to represent ESC RCC.^102^ Another recently described provisional renal entity, designated ‘xanthomatous giant cell RCC associated with TSC2 mutations’ also likely falls within the spectrum of ESC RCC, owing to its morphological, IHC and molecular similarities with ESC RCC (Figure 6A,B).^103^, ^104^ Although some studies have highlighted the morphological and IHC similarities (such as CK20 and cathepsin K reactivity) with TFEB‐altered RCC, *TSC* mutations have not been found in TFEB‐altered RCC, except in one TFEB‐amplified RCC, likely as a passenger mutation.^105^ TFEB‐amplified RCCs however exhibit more aggressive behaviour and the distinction from ESC RCC, largely an indolent tumour, is important.^106^

ESC RCC is typically a sporadic and solitary tumour found in patients of broad age range, with marked female predilection.^2^, ^68^, ^100^, ^101^ About 5% of reported cases have been found in patients with TSC and some ESC RCCs in TSC have been described under different names before its recognition.^47^, ^107^ ESC RCCs have also been found to occur in the same kidney with LOT and EVT in patients with TSC^34^, ^47^, ^107^ and have been found as well in patients with end‐stage renal disease.^33^, ^34^ ESC RCC is typically an indolent tumour in the majority of cases, but examples with metastases have been reported in about 5–10% of cases, which were typically of larger size.^108^, ^109^, ^110^

ESC RCC grossly shows solid and cystic components or very rarely only microcysts are found microscopically in a solid tumour.^99^, ^100^, ^101^ The solid areas are composed of eosinophilic cells exhibiting diffuse or compact nested growth, but other architectural patterns, including focal papillary growth, clear cell areas, focal insular or tubular growth, and clusters of multinucleated cells may also be present (Figure 6A,B).^100^, ^101^ The cysts are lined by eosinophilic cells often showing a hobnailed pattern. One of the most consistent and helpful diagnostic features is the presence of basophilic to amphiphilic coarse cytoplasmic granules (stippling), sometimes mimicking ‘leishmania bodies’ which can be easily appreciated on microscopy (Figure 6C).^99^, ^100^, ^101^ On electron microscopy, the coarse cytoplasmic granularity stems from the aggregates of rough endoplasmic reticulum and granular cytoplasmic material.^100^

ESC RCC typically shows diffuse or focal CK20 expression on IHC (Figure 6D) which is perhaps the most useful IHC marker; however, a minority of cases (about 10%–15%) may be CK20 negative.^2^, ^100^, ^101^ CK7 is, however, consistently negative in ESC RCC^2^, ^100^, ^101^ resulting in a CK20+/CK7− IHC profile. Cathepsin K reactivity has been reported to be at least focally positive,^108^ melan A can be either negative or positive in only rare cells, while GPNMB is uniformly positive (Figure 6E,F).^40^, ^102^ Vimentin is typically positive, in contrast to most oncocytic renal tumours, such as LOT, EVT, RO, and ChRCC.^100^, ^101^ ESC RCC is negative for CD117 (KIT), GATA3, and L1CAM. Of note, TRIM63 RNA in situ hybridization that has been proposed as a novel diagnostic biomarker for TFE3 and TFEB‐altered RCCs^111^ has also been reported positive in 38.5% of ESC RCCs.^112^

ESC RCC demonstrates somatic biallelic loss mostly in *TSC2* and less often in *TSC1*, and such losses represent clonal events, resulting in activation of the mTORC1.^2^, ^18^, ^108^, ^113^, ^114^ Despite the commonality of the underlying TSC/MTOR pathway alterations with other tumours, such as EVT and LOT, ESC RCC forms an independent genomic cluster, which is also distinct from other common renal tumours, such as clear cell RCC, ChRCC, and RO.^20^, ^104^

## Anaplastic Lymphoma Kinase‐Rearranged Renal Cell Carcinoma (ALK‐Rearranged RCC)

*ALK*‐rearranged RCC was first described in 2011^115^, ^116^ and it was recognized in the WHO 2022 classification as a new renal entity, characterized by an *ALK* gene fusion.^117^ *ALK* is located on chromosome 2p23 and partners with various genes, including *VCL, HOOK1, STRN, TPM3, EML4*, *PLEKHA7*, *CLIP1, KIF5B, KIAA1217, SLIT1, TPM1*, and *NUMA1*,^2^, ^118^, ^119^, ^120^, ^121^ resulting in aberrant *ALK* activation. *ALK*‐rearranged RCC has been documented in patients of a wide age range, including the paediatric population^122^ and of diverse racial backgrounds.^2^, ^117^, ^118^ *ALK‐*rearranged RCC with *VCL* fusion has often been found in patients with sickle cell trait.^119^, ^123^, ^124^ The majority of ALK‐rearranged RCCs are indolent, but an adverse clinical course with metastatic disease has been reported in about 20%–30% of patients^2^, ^118^

**Figure 7:**
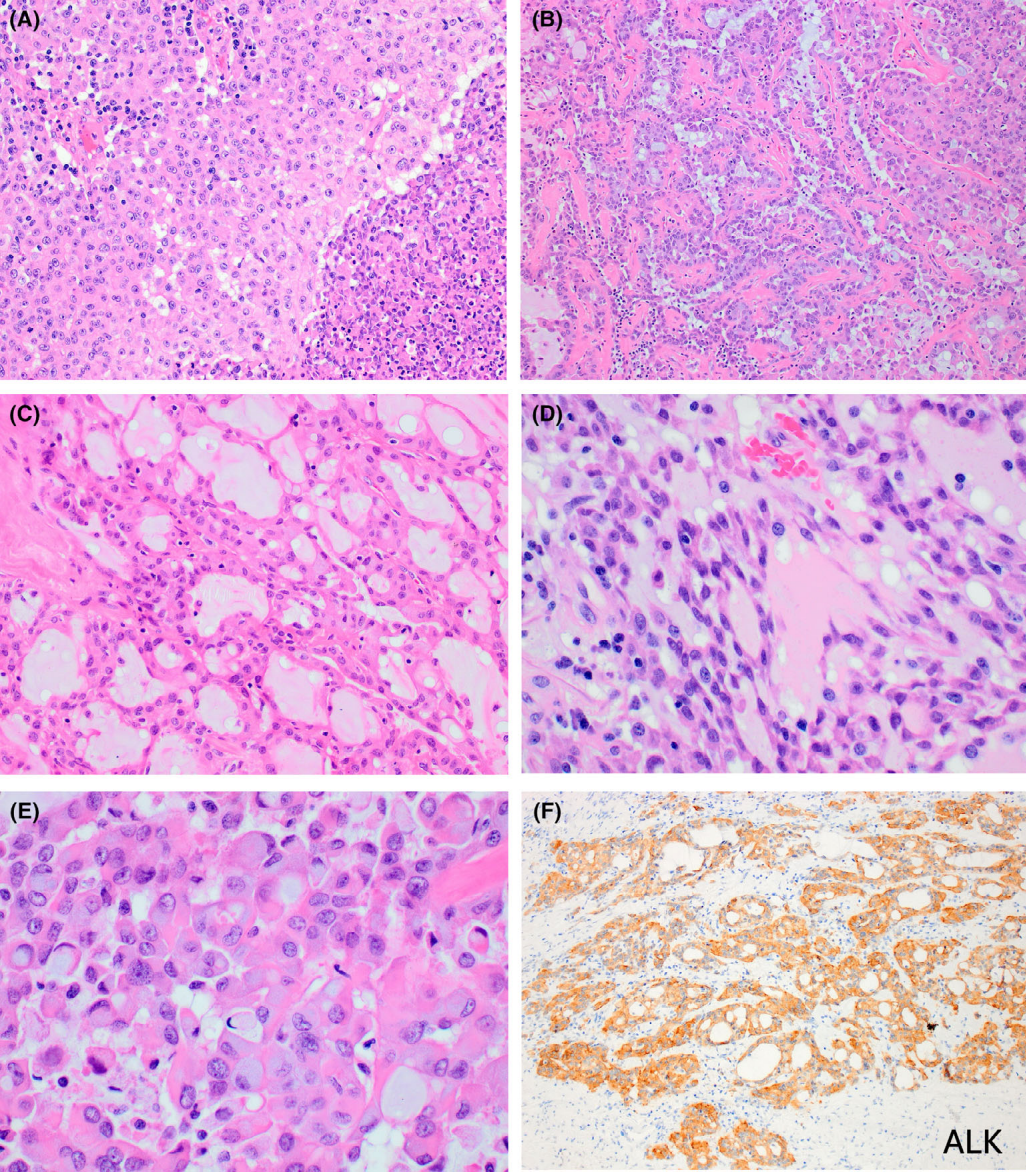
*ALK*‐rearranged RCC. (**A‐E**) *ALK*‐ rearranged RCC shows diverse morphology that includes a multitude of growth patterns, including solid (**A**), papillary (**B**), tubular/tubulocystic (**C**), trabecular, cribriform, “metanephric adenoma‐like”, “mucinous tubular and spindle cell RCC‐like” (**D**), and signet‐ring/single cell (**E**). Mucinous component, both intracellular and interstitial, is a common finding, and should raise suspicion for *ALK*‐rearranged RCC. (**F**) ALK protein expression on IHC can be used to screen for *ALK*‐ rearranged RCC.

*ALK*‐ rearranged RCC is a solitary tumour with diverse morphology that includes a multitude of growth patterns, including solid, papillary, tubular, tubulocystic, trabecular, cribriform, signet‐ring/single cell, ‘mucinous tubular and spindle cell RCC‐like’ and ‘metanephric adenoma‐like’ (Figure 7A–E).^2^, ^118^ Mucinous component, both intracellular and interstitial, is a common finding, and should raise suspicion for *ALK*‐rearranged RCC.^2^, ^118^ The variety of observed morphological patterns is likely related to the different gene fusion partners and may invoke a broad differential.

*ALK*‐rearranged RCC is consistently reactive for ALK on IHC, in particular using the monoclonal antibodies 5A4 and D5F3 (Figure 7F).^118^, ^119^, ^122^, ^125^ The remaining IHC profile of *ALK*‐rearranged RCC is variable, but includes reactivity for PAX8 and vimentin, and retained FH and INI1, the last two being useful in differentiating it from FH‐deficient RCC and SMARCB1‐deficient renal medullary carcinoma, respectively. Molecular diagnosis of *ALK* rearrangement can be established using break‐apart FISH or NGS. Due to its varied morphology, screening for ALK by IHC or FISH and other molecular methods should be performed in any difficult‐to‐classify renal eosinophilic tumour showing variable growth patterns or containing intracellular or interstitial mucin.^118^

Given the clinical availability of ALK inhibitor targeted therapies, it is important not to miss the diagnosis of *ALK*‐rearranged RCC in practice.^126^, ^127^, ^128^ Several reports have documented a strong response to ALK inhibitors in metastatic *ALK‐*rearranged RCC and resistance to other common RCC treatments.^129^, ^130^, ^131^

## Fumarate Hydratase‐Deficient Renal Cell Carcinoma (FH‐Deficient RCC)

**Figure 8:**
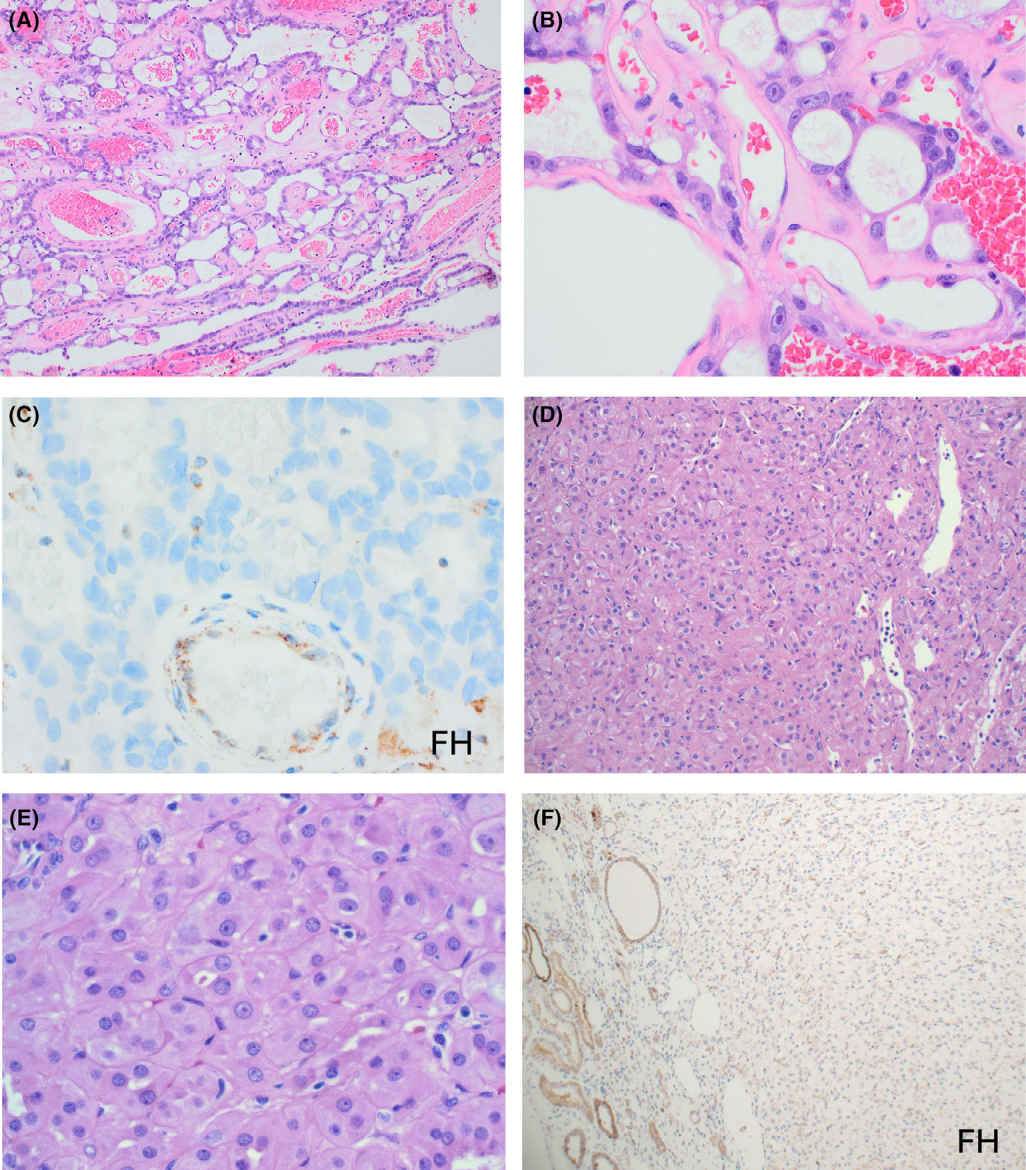
FH‐deficient RCC. (**A**) FH‐deficient RCC typically demonstrates various architectural patterns, including tubulocystic and papillary (bottom). (**B**) FH‐deficient RCC typically exhibits at least focal eosinophilic macronucleoli with perinucleolar halos, which are, however, not specific and may be found in other high‐grade RCCs. (**C**) Negative staining for FH in the tumoral cells is a specific IHC test for this entity. (**D, F**) Rare “low‐grade oncocytic” FH‐deficient RCC has been reported, showing low‐grade nuclei and oncocytic cytoplasm with variable cytoplasmic vacuoles, mimicking SDH‐deficient RCC (**D**, **E**). Some have shown metastatic potential despite the low‐grade morphology. The IHC profile of low‐grade FH‐deficient RCCs is identical to their high‐grade counterparts, with a lack of reactivity for FH (**F**).

FH‐deficient renal cell carcinoma (FH‐deficient RCC), previously referred to as hereditary leiomyomatosis and renal cell carcinoma (HLRCC) syndrome‐associated RCC, is a distinct subtype of RCC, characterized by biallelic inactivation of the *FH* gene. The *FH* gene (located on chromosome 1q43) encodes FH, a key enzyme in the Krebs cycle, and its loss disrupts cellular metabolism and promotes tumorigenesis.^132^, ^133^, ^134^, ^135^ Alterations in the *FH* gene can be either germline, as seen in the HLRCC syndrome, or rarely sporadic and somatic.^132^, ^133^, ^134^, ^135^, ^136^ FH‐deficient RCCs are typically aggressive tumours presenting at high stage.^137^, ^138^, ^139^, ^140^, ^141^, ^142^ They often exhibit eosinophilic, high‐grade papillary morphology, frequently admixed with other architectural patterns, including tubular, tubulocystic, solid/sarcomatoid, intracystic, cribriform, and sieve‐like (Figure 8A,B).^137^, ^141^, ^143^, ^144^, ^145^ FH‐deficient RCC typically exhibits at least focal eosinophilic macronucleoli, often with perinucleolar halos, which are however not specific for FH‐deficient RCC and may be found in other high‐grade RCCs.^137^, ^145^ In the past, FH‐deficient RCCs were likely misdiagnosed as type 2 papillary RCCs, tubulocystic RCC with dedifferentiation, collecting duct RCCs, and high‐grade unclassified RCCs.^137^, ^138^, ^145^

FH‐deficient RCC is characterized by a loss of FH expression on IHC (Figure 8C,F), along with s‐2‐succinocysteine (2SC) expression, which is both nuclear and cytoplasmic.^137^, ^140^, ^146^ Such dual nuclear and cytoplasmic 2SC staining increased the sensitivity and specificity to 100% and 91%, respectively.^146^ The FH loss on IHC is considered specific but less sensitive^119^, ^137^ while 2SC has higher sensitivity but is less specific^146^; a combination of both resulting in FH−/2SC+ profile optimizes the diagnostic potential.^137^, ^146^ Currently, WHO 2022 recommends either FH−/2SC+ve profile or molecular confirmation to establish a diagnosis of FH‐deficient RCC.^147^ Heterogeneous loss or focal and weak expression for FH has also been found in some FH‐deficient RCCs with missense mutations.^148^ Recently, an aldo‐keto reductase family 1 member B10 (AKR1B10), used as an IHC biomarker, has been found to have a 100% sensitivity and better specificity (91.4% vs. 88.9%) than 2SC.^149^ Other IHC stains are variable and non‐specific.

An increasing number of so‐called ‘low‐grade oncocytic’ FH‐deficient RCC have been reported.^58^, ^141^, ^150^, ^151^, ^152^ These are eosinophilic tumours with solid and nested architecture but with low‐grade nuclei and oncocytic cytoplasm with variable cytoplasmic vacuoles, mimicking SDH‐deficient RCC (Figure 8D,E). Some ‘low‐grade oncocytic’ FH‐deficient RCC displayed pure low‐grade morphology, while some showed focal high‐grade areas. There is a debate about the suitability of the ‘low‐grade’ designation for this FH‐deficient RCC group, because although most of them are indolent, some have shown metastatic potential.^153^, ^154^ Such ‘low‐grade oncocytic’ FH‐deficient RCCs broaden the spectrum of this entity and should also be considered in the differential of any low‐grade oncocytic neoplasm, particularly on biopsy. The IHC profile and the molecular profile of low‐grade FH‐deficient RCCs are identical to their high‐grade counterparts (Figure 8F).

Finally, it is important to correctly diagnose FH‐deficient RCC because it necessitates genetic counselling and surveillance for the patient and the family members owing to its hereditary potential. More recently, there was an emergence of a novel therapy for FH‐deficient RCC that includes bevacizumab, an anti‐VEGF monoclonal antibody, and erlotinib, an anti‐EGFR tyrosine kinase inhibitor.^155^, ^156^ Such dual treatment has shown superior response in FH‐deficient RCC compared to sporadic papillary RCC.^155^, ^156^ Early clinical trials using a combination of immune checkpoint inhibitor therapy with tyrosine kinase inhibitor therapy have also shown some promise in the treatment of FH‐deficient RCC.^157^ The potential immune therapy response in FH‐deficient RCC may be related to its enhanced immune signature with increased PDL1 expression.^158^

## Papillary Renal Neoplasm of Reverse Polarity (PRNRP)

**Figure 9:**
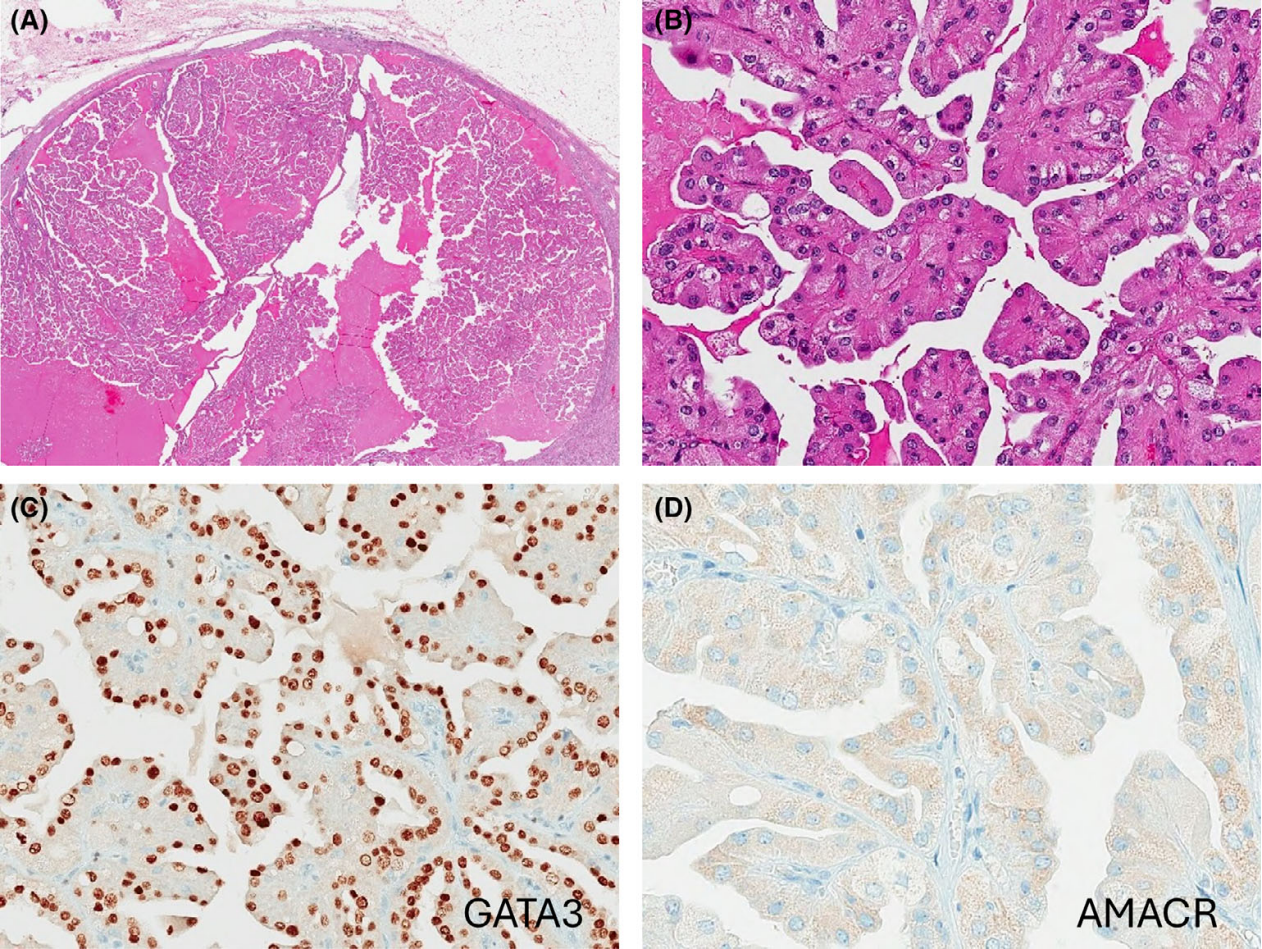
Papillary renal neoplasm of reversed polarity (PRNRP). (**A**) PRNRP is a non‐encapsulated, small tumour that typically measures <15 mm in size. (**B**) It demonstrates delicate papillae lined by a single layer of oncocytic cells with low‐grade nuclei, showing a linear arrangement along the lumina and opposite the base (*‘reverse polarity’*). (**C**, **D**) PRNRP is uniformly positive for GATA3 (**C**), while it is usually negative for AMACR (**D**).

‘Papillary RCC with oncocytic cells and nonoverlapping low‐grade nuclei’,^159^ likely represents the seminal description of this entity in 2008. It was further characterized in 2017^160^ and 2019,^161^ when the name ‘papillary renal neoplasm of reverse polarity (PRNRP)’ was proposed. Morphologically, it shows a delicate papillary growth, and the papillae are lined by a single layer of oncocytic cells with low‐grade nuclei (equivalent to WHO grade 1–2), with a linear arrangement along the luminal aspect and away from the base (*‘reverse polarity’*) (Figure 9A,B). PRNRP represented about 4% of all papillary RCCs and 0.5% of all renal tumours in one longitudinal institutional series.^162^ To date, few hundred cases have been documented in the literature without a single report of malignant behaviour.^161^, ^163^, ^164^, ^165^, ^166^, ^167^

PRNRP is usually smaller in size than conventional papillary RCC^160^, ^161^ and it is often <15 mm, which is the cutoff size for papillary adenoma.^168^ PRNRP typically lacks necrosis, high‐grade morphology, and it is confined to the kidney.^161^, ^163^, ^164^, ^165^ The majority of PRNRPs show *KRAS* mutations that tend to be clustered in exon 2‐codon 12.^163^, ^169^ On IHC, PRNRP is positive for GATA3, L1CAM, high molecular weight keratin (HMWK) and CK7, while it is generally negative for vimentin and α‐methylacyl‐CoA racemase (AMACR) (Figure 9C,D).^160^, ^161^, ^167^, ^170^, ^171^

A growing body of evidence indicates that PRNRP is a separate entity from papillary RCC. The IHC pattern of positive GATA3, L1CAM, HMWK, along with a lack of reactivity for vimentin and AMACR, suggests a distal nephron origin, in contrast to papillary RCC, thought to arise from the proximal tubules.^172^ PRNRP also shares a similar gene expression profile with the distal renal tubules,^173^ and exhibits different expression profiles of noncoding RNA from papillary RCC.^174^ Several recent studies using array‐based comparative genomic hybridization (aCGH) and single‐nucleotide polymorphism (SNP) microarrays found that gains in chromosomes 7 and 17 and a loss of chromosome Y are much less common or absent in PRNRP than in papillary RCC.^166^, ^167^, ^175^, ^176^

Because PRNRP is an indolent tumour, it is important to distinguish it from eosinophilic papillary RCC, which, in contrast, may have an aggressive behaviour and high‐grade morphology.^177^ *KRAS* mutations are, however, not specific for PRNRP, and a subset of PRNRPs do not harbour *KRAS* mutations (about 10%–30%).^162^, ^163^, ^167^ On the other hand, less than 1% of all papillary RCCs (even high‐grade ones) exhibit *KRAS* mutations,^163^, ^178^ which suggests that these alterations are of diagnostic significance only in the context of appropriate morphology and IHC profile. In sum, the distinct morphology, unique IHC and molecular profiles, along with the uniformly indolent clinical behaviour, distinguish PRNRP from papillary RCC and support the notion that it represents a separate entity.

## Tubulocystic Renal Cell Carcinoma (TC‐RCC)

**Figure 10:**
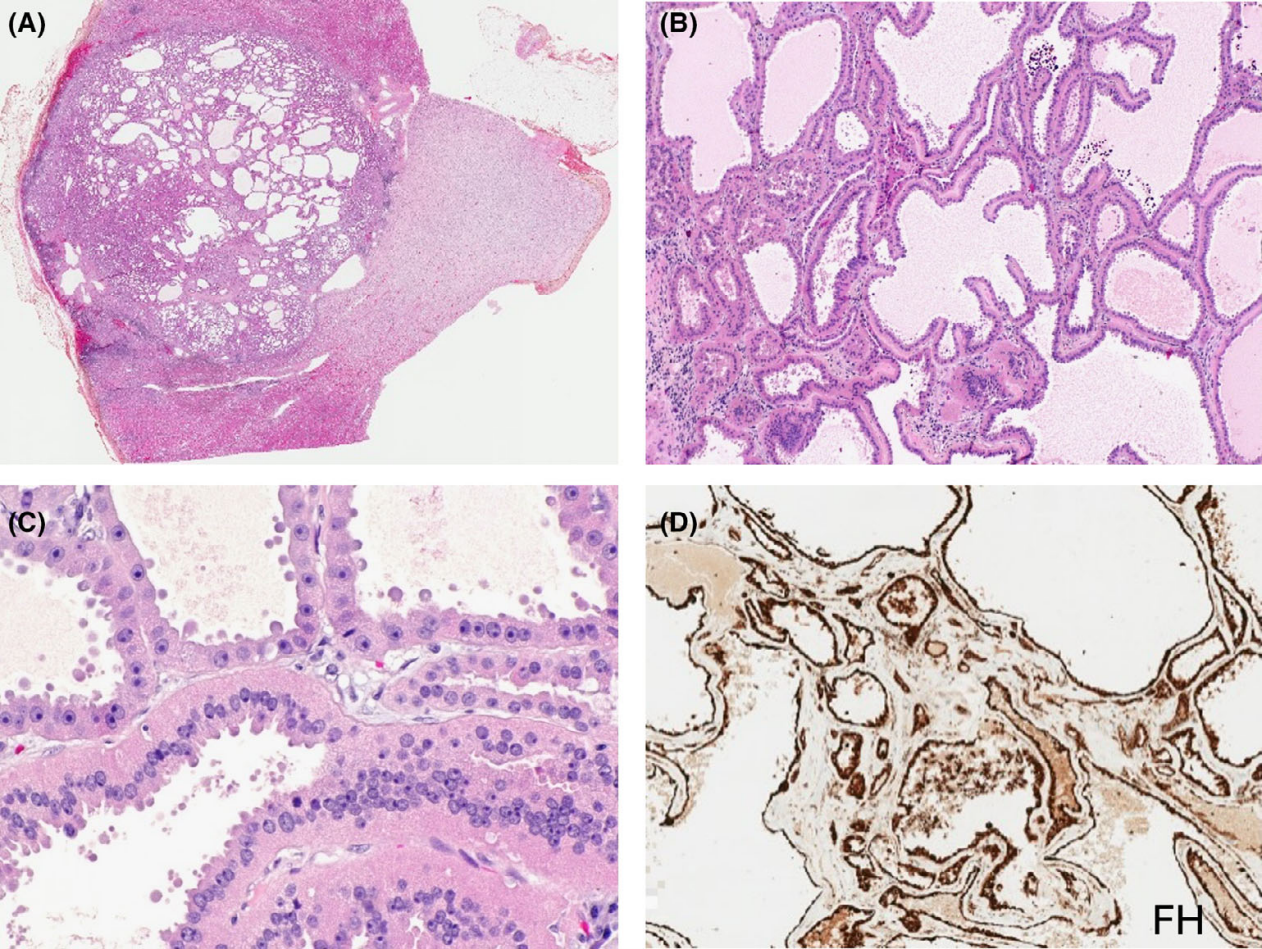
Tubulocystic (TC)‐RCC. (**A**, **B**) TC‐RCC typically shows pure tubulocystic morphology with minimal intervening stroma. (**C**) The cells lining the tubules and the cysts are eosinophilic and show prominent nucleoli with delicate apical snouts. (**D**) TC‐RCC shows retained expression of FH and lack of reactivity for 2SC (not shown), which helps rule out FH‐deficient RCC that often shows tubulocystic growth.

TC‐RCC is a rare tumour representing <1% of all RCCs that was first proposed as a separate renal entity in 2009.^179^ TC‐RCC typically exhibits a distinct bubble wrap gross appearance and an exclusive tubulocystic morphology with minimal intervening fibrotic/hypocellular stroma.^179^ The cells lining the tubules and the cysts are eosinophilic, arranged in a single layer of flattened, cuboidal or hobnailed cells with prominent nucleoli (equivalent to WHO Grade 3), often showing delicate apical snouts (Figure 10A–C).^1^ However, TC‐RCC carcinoma should not be graded, because it is generally considered an indolent tumour. Of note, the seminal study on TC‐RCC reported disease progression in 2 of 31 TC‐RCCs.^179^ Recently, Xiao *et al*. reported 28 cases of pure TC‐RCC, all with an indolent clinical outcome; of note, they found that 96% of pure TC‐RCCs were associated with end‐stage kidney disease.^180^ The ability to diagnose TC‐RCC has also been explored in imaging studies, where such cases showed multilocular cystic lesions (Bosniak classification II‐IV).^181^, ^182^

Importantly, tubulocystic growth pattern can be seen in other tumours, and for example, it has been found as a common pattern in FH‐deficient RCC.^137^, ^140^, ^181^ Although there are reports of ‘tubulocystic carcinoma’ admixed with other morphologies, including papillary, solid, cribriform, sarcomatoid and poorly differentiated^183^, ^184^, ^185^ demonstrating aggressive behaviour and poor outcome, these ‘non‐pure tubulocystic carcinomas’ represent likely unrecognized FH‐deficient RCCs.^140^ For example, Smith *et al*. found that >80% of ‘tubulocystic carcinomas with poorly differentiated foci’ exhibit *FH* aberrations.^140^ On IHC, TC‐RCC typically stains for AMACR, vimentin, CK7 and CD10,^186^, ^187^, ^188^ with retained expression of FH (Figure 10D) and lack of reactivity for 2SC. Thus, it is essential to exclude FH‐deficient RCC before rendering a diagnosis of TC‐RCC, given the clinical and prognostic implications.^1^

The reported molecular makeup of previously documented TC‐RCCs has also been influenced by the inclusion of non‐pure TC‐RCCs. Pure TC‐RCCs, however, exhibit losses of chromosomes 9, Y and gains of chromosome 17.^189^, ^190^ Some pure TC‐RCCs also harbour mutations in chromatin modifying genes *KMT2C* and *KDM5C*, but so far, no specific gene mutation has been found.^189^ Recently, the *NSD2* gene, an H3K36‐specific di‐methyltransferase, has been found to facilitate the development of TC‐RCC from polycystic kidney disease in an animal mouse model, through the integrin/FAK/AKT pathway.^191^ TRIM63 RNA, a biomarker classically overexpressed in the MiT family of RCCs, was also overexpressed in ~60% of a limited number of studied TC‐RCCs.^112^

## Thyroid‐Like Follicular Carcinoma of Kidney (TLFCK)

The first case report of thyroid‐like follicular carcinoma of kidney (TLFCK) was published in 2006^192^ and the first series of six cases was reported in 2009.^193^ TLFCK is an extremely rare renal tumour that was considered an emerging renal entity by the GUPS consensus^2^ and was also listed in the 2022 WHO classification as an ‘emerging entity’.^3^ However, no consistent IHC profile and molecular genetic changes have been identified so far, despite the morphological similarities in about 60 reported cases, mostly as individual case reports.(reviewed in^2^, ^52^). It is likely that some of the reported cases of TLFCK represent other entities, such as papillary RCC variants,^2^, ^194^, ^195^, ^196^, ^197^ a recently described atrophic kidney‐like lesion^198^, ^199^, ^200^ or metastatic thyroid carcinoma with unidentified primary. TLFCK is slightly more frequent in females and has been found in patients of broad age range^52^

**Figure 11:**
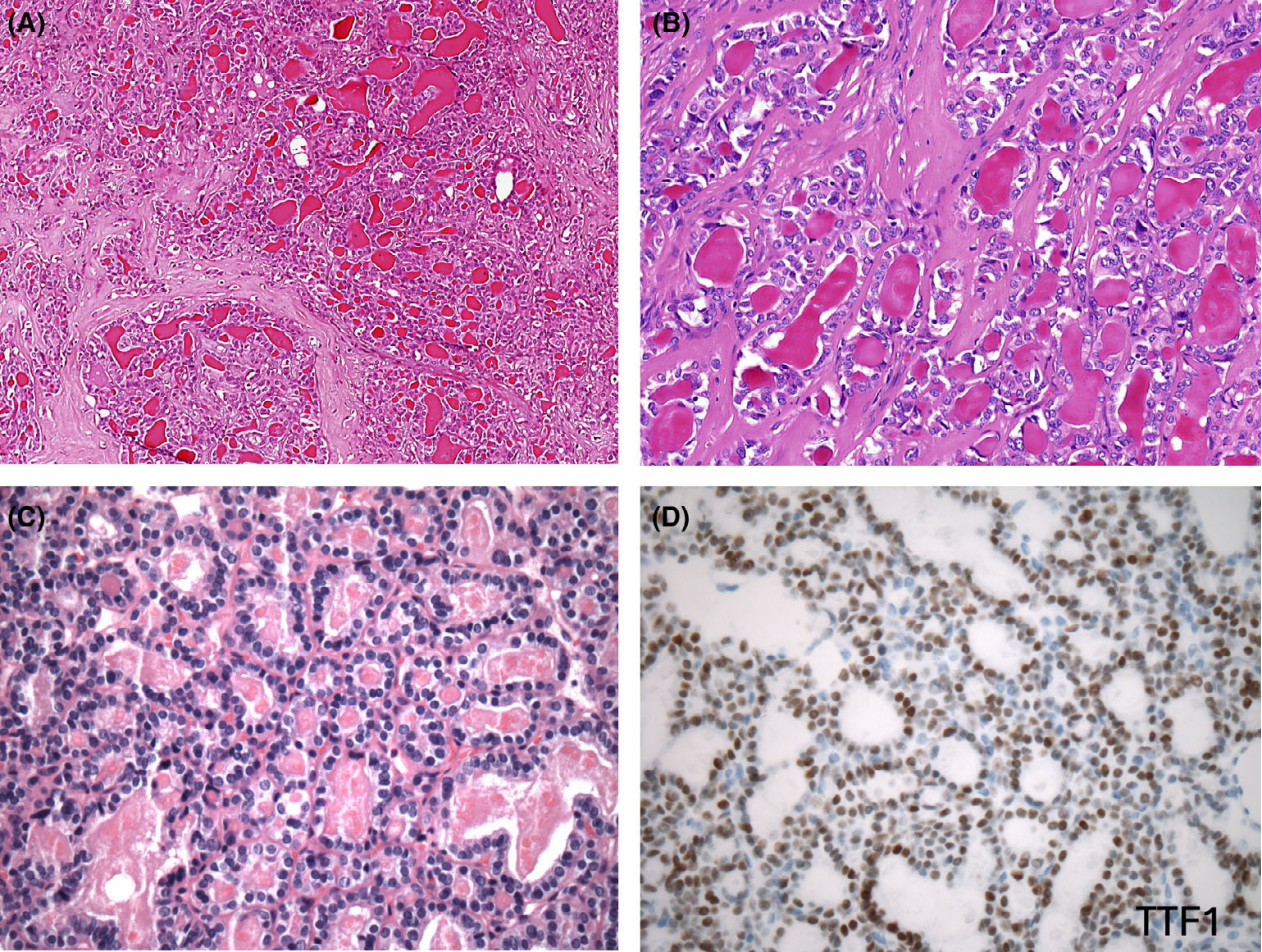
Thyroid‐like follicular carcinoma of kidney (TLFCK). (**A**, **B**) TLFCK shows a follicular pattern resembling thyroid‐like follicular carcinoma; the follicles are lined by eosinophilic cells and contain dense eosinophilic colloid. (**C**, **D**) Metastatic thyroid follicular carcinoma to the kidney is the main differential, as it is morphologically virtually indistinguishable from TFLCK (**C**); TTF1 (**D**) and thyroglobulin are typically positive on IHC in metastatic thyroid follicular carcinoma, while they are both negative in TLFCK (both are however positive for PAX8).

On microscopy, TLFCK exhibits a follicular growth pattern resembling follicular carcinoma of the thyroid (Figure 11A,B). The follicles are filled with dense colloid‐like material and are typically arranged back‐to‐back, but may show branching and confluent growth, resulting in a focal papillary pattern, as well as microcalcifications. They are lined by eosinophilic cells that have uniform nuclei (WHO grade 2–3). Importantly, TLFCKs are negative for TTF1 and thyroglobulin, distinguishing them from metastatic thyroid follicular carcinoma, the main differential diagnosis (Figure 11C,D). TLFCK is typically positive for PAX8, as is thyroid follicular carcinoma, but the remaining reported IHC profile has been variable.

TLFCK was an indolent tumour in great majority of reported cases, but several patients have been documented with metastases to regional lymph nodes, lung, retroperitoneum, skull, and meninges.^201^, ^202^, ^203^, ^204^, ^205^, ^206^ There are also three patients reported with TLFCK accompanied by sarcomatoid differentiation showing sheets and fascicles of round to spindle cells; all three presented with metastases, two of whom died of disease, and one was found to have a *EWSR1*::*PATZ1* fusion.^207^, ^208^, ^209^ This is notable, because *EWSR1*::*PATZ1* fusion has also been found in some round to spindle cell sarcomas occurring at various locations (for example, deep soft tissue of chest wall and abdomen, head/neck, and central nervous system); these have been classified as ‘round cell sarcomas with EWSR1–non‐ETS fusions’ in the 2020 WHO Classification of Soft Tissue and Bone Tumours.^210^ In addition, *EWSR1*::*PATZ1* fusion has been recently identified in 3 of 8 patients with indolent TFFCK, but without sarcomatoid change.^211^ Whether this first documented recurrent gene fusion in TLFCK is indeed specific for this entity, or at least for a subset of TLFCK, warrants further validation.^211^ To date, no other consistent chromosomal copy number changes nor recurrent genetic alterations have been identified in TLFCK.

## Author contributions

K. Trpkov designed and drafted the study. F. Siadat provided editing, critical feedback, and drafted the figures. R. Saleeb drafted parts of the study and provided editing and critical feedback.

## Funding information

The authors received no financial support for the research, authorship, and/or publication of this article.

## Conflict of interest

Authors declared no potential conflicts of interest with respect to the research, authorship, and/or publication of this article.

## Data Availability

The content of the article is based on data obtained by literature review (i.e. publicly available data).
